# Transcript profiling of two alfalfa genotypes with contrasting cell wall composition in stems using a cross-species platform: optimizing analysis by masking biased probes

**DOI:** 10.1186/1471-2164-11-323

**Published:** 2010-05-24

**Authors:** S Samuel Yang, Wayne Wenzhong Xu, Mesfin Tesfaye, JoAnn FS Lamb, Hans-Joachim G Jung, Kathryn A VandenBosch, Carroll P Vance, John W Gronwald

**Affiliations:** 1 USDA-Agricultural Research Service, Plant Science Research Unit, St. Paul, MN 55108, USA; 2 Supercomputing Institute for Advanced Computational Research, University of Minnesota, Minneapolis, MN 55455, USA; 3 Department of Plant Biology, University of Minnesota, St. Paul, MN 55108, USA; 4 Department of Agronomy and Plant Genetics, University of Minnesota, St. Paul, MN 55108, USA

## Abstract

**Background:**

The GeneChip^® ^*Medicago *Genome Array, developed for *Medicago truncatula*, is a suitable platform for transcript profiling in tetraploid alfalfa [*Medicago sativa *(L.) subsp. *sativa*]. However, previous research involving cross-species hybridization (CSH) has shown that sequence variation between two species can bias transcript profiling by decreasing sensitivity (number of expressed genes detected) and the accuracy of measuring fold-differences in gene expression.

**Results:**

Transcript profiling using the *Medicago *GeneChip^® ^was conducted with elongating stem (ES) and post-elongation stem (PES) internodes from alfalfa genotypes 252 and 1283 that differ in stem cell wall concentrations of cellulose and lignin. A protocol was developed that masked probes targeting inter-species variable (ISV) regions of alfalfa transcripts. A probe signal intensity threshold was selected that optimized both sensitivity and accuracy. After masking for both ISV regions and previously identified single-feature polymorphisms (SFPs), the number of differentially expressed genes between the two genotypes in both ES and PES internodes was approximately 2-fold greater than the number detected prior to masking. Regulatory genes, including transcription factor and receptor kinase genes that may play a role in development of secondary xylem, were significantly over-represented among genes up-regulated in 252 PES internodes compared to 1283 PES internodes. Several cell wall-related genes were also up-regulated in genotype 252 PES internodes. Real-time quantitative RT-PCR of differentially expressed regulatory and cell wall-related genes demonstrated increased sensitivity and accuracy after masking for both ISV regions and SFPs. Over 1,000 genes that were differentially expressed in ES and PES internodes of genotypes 252 and 1283 were mapped onto putative orthologous loci on *M. truncatula *chromosomes. Clustering simulation analysis of the differentially expressed genes suggested co-expression of some neighbouring genes on *Medicago *chromosomes.

**Conclusions:**

The problems associated with transcript profiling in alfalfa stems using the *Medicago *GeneChip as a CSH platform were mitigated by masking probes targeting ISV regions and SFPs. Using this masking protocol resulted in the identification of numerous candidate genes that may contribute to differences in cell wall concentration and composition of stems of two alfalfa genotypes.

## Background

Alfalfa [*Medicago sativa *(L.) subsp. *sativa*] is the most widely cultivated forage legume in the world [1] and the fourth most widely grown crop in the United States [2]. In 2008, over 60 million metric tons of alfalfa dry hay with a value of over $10 billion were harvested from over 8.5 million hectares in the US [3]. In addition to being a valuable forage crop for livestock, alfalfa has considerable potential as a sustainable, cellulosic feedstock for ethanol production [2]. Alfalfa is a relatively high biomass crop that also provides environmental benefits [2]. For example, alfalfa improves soil and water quality, promotes wildlife diversity and provides its own nitrogen fertilizer through symbiotic nitrogen fixation [2,4-6].

A promising strategy for developing alfalfa as a cellulosic ethanol crop involves separating leaves and stems following harvest [2]. The leaves would be used as a protein supplement for livestock while the stems would be used to produce ethanol. Our research has focused on selecting for large stem, non-lodging, biomass-type alfalfa germplasm and developing management strategies to optimize biomass yield. To date, these efforts have resulted in a 40% increase in total biomass and a doubling of theoretical ethanol yield [7]. We have also initiated research to modify the composition of alfalfa stem cell walls via a transgenic approach. The efficiency of ethanol production from cellulosic biomass is positively correlated with cellulose content but negatively correlated with lignin content [8,9]. Thus, the value of alfalfa as a cellulosic feedstock would be enhanced by developing new alfalfa varieties that have increased cellulose and decreased lignin in stem cell walls [8,9]. To facilitate the identification of key genes regulating cell wall composition, we selected alfalfa germplasm (genotypes 252 and 1283) that exhibit significant differences in lignin and cellulose concentrations in stem cell walls [10]. On a dry matter basis, stem cellulose and Klason lignin concentrations of plants at flowering are significantly higher in genotype 252 compared to genotype 1283 (302 gkg^-1 ^vs. 257 gkg^-1 ^for cellulose and 117 gkg^-1 ^vs. 98 gkg^-1^for Klason lignin, respectively).

A high-density oligonucleotide microarray is not yet available for global transcript profiling in alfalfa. However, the GeneChip^® ^*Medicago *Genome Array is available. This GeneChip contains a total of 52,796 *Medicago *probe sets designed from 50,900 and 1,896 sequences from *M. truncatula *and alfalfa, respectively. Each probe set in the GeneChip consists of 11 perfect match (PM) and 11 mismatch (MM) 25-mer probes. An underlying assumption when using microarrays for cross-species hybridization (CSH) is that the level of sequence homology among genes of closely-related species is significant enough to enable detection by probes originally designed for their orthologs. Previous research indicated that the *Medicago *GeneChip^® ^is a suitable cross-species platform for transcript profiling in alfalfa [11,12]. In large part, this is because there is a significant level of gene homology. For example, a previous study reported that DNA sequence identity was 93% or greater between protein coding regions of selected homologous genes in alfalfa and *M. truncatula *[11]. However, in previous research using the *Medicago *GeneChip^® ^for transcript profiling in alfalfa tissues, we observed decreased sensitivity (number of genes detected) and decreased accuracy in measuring fold-changes in gene expression compared to results obtained with *M. truncatula *tissues [11,12].

Numerous studies, conducted with both animals and plants, have reported transcript profiling involving CSH to DNA microarrays of a closely-related species [13-32]. In a number of these studies, electronic masking was used to remove biased probes prior to microarray data analysis. For example, Ranz et al. [18] introduced a probe selection method based on genomic DNA hybridizations of the target and non-target species to the GeneChip. This approach has been used for CSH studies involving plant species [19,27,30]. However, a recent CSH study involving *Xenopus *species questioned the reliability of this method for selecting unbiased probes [32]. Transcript profiling in non-human primates using the human GeneChip for CSH was optimized by identifying inter-species conserved probe sets [26]. These probe sets were identified by aligning expressed sequence tags (ESTs) in non-human primate with probe sequences on the Affymetrix human GeneChip^® ^platform. However, this approach is not feasible for species with limited sequence information such as alfalfa. In a study using the human GeneChip as a cross-species platform to measure gene expression in heart and liver tissues of non-human mammals (e.g. cattle, pig, dog, mouse), Ji et al. [21] developed a protocol to selectively mask poorly hybridized probes using the match/mismatch feature of the GeneChip. To evaluate whether masking improved the accuracy of measuring gene expression, it was hypothesized that different organs (heart, liver) of humans and non-human mammals have similar gene expression patterns. After masking low intensity probes in the microarray data of the cross-species, Ji et al. [21] found a linear correlation (r = 0.93) for Ln(heart/liver) values between human and mouse GeneChip data. These authors concluded that comparisons of gene expression patterns in defined tissues of related species could be used to optimize CSH studies involving other mammals or plants.

In earlier research, we examined gene expression at two stages of stem development for alfalfa and *Medicago truncatula *[12]. In both species, transcript profiling was conducted in elongating stem internodes (ES) and post-elongation stem internodes (PES). Genes associated with primary cell wall development were preferentially expressed in ES internodes while genes associated with secondary xylem development were enriched in PES internodes. The objective of this study was to identify genes that are differentially expressed in ES and PES internodes of alfalfa genotypes 252 and 1283 using the GeneChip^® ^*Medicago *Genome Array as a cross-species platform. To optimize cross-species hybridization analysis, we developed a protocol for masking probes targeting inter-species variable (ISV) regions. After masking for ISV regions and single-feature polymorphisms (SFPs) previously detected in genotypes 252 and 1283 [10], we identified numerous genes that were differentially expressed in ES and PES internodes of the two genotypes.

## Results and discussion

### Masking probes targeting inter-species variable regions

As a preliminary analysis of sequence divergence between orthologous genes of *Medicago truncatula *and *Medicago sativa *(alfalfa), we blasted 550,074 *M. truncatula *probe sequences (25-mer) on the GeneChip^® ^Medicago Genome Array against the 12,072 alfalfa expressed sequence tag (EST) sequences that are currently available from the public database (e-value cut-off = 0.001, minimum nucleotide alignment length = 20). A total of 21,176 *M. truncatula *probe sequences had alfalfa EST hits and 14,960 of them (~70%) showed at least one base mismatch (data not shown). These results suggested that masking ISV regions would optimize transcript profiling when using the *Medicago *GeneChip^® ^as a cross-species platform for measuring gene expression in alfalfa.

**Figure 1:**
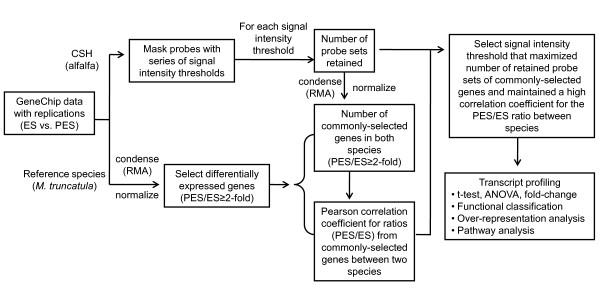
**Work-flow diagram for masking probes targeting ISV regions**. Commonly-selected genes are defined as genes exhibiting at least a 2-fold difference in hybridization intensity expression ratio between PES and ES internodes (PES vs. ES, ≥2-fold difference) of both alfalfa and *M. truncatula*. RMA, Robust Multi-array Average; ES, elongating stem; PES, post-elongation stem; CSH, cross-species hybridization.

We developed a protocol to mask probes targeting ISV regions of the probe sets expressed in ES and PES internodes of alfalfa genotypes 252 and 1283. A work-flow diagram of the protocol used to mask probes targeting ISV regions is shown in Figure 1. For each tissue sample, three biological replicates were collected producing a total of 12 data points per probe (2 tissues × 2 genotypes × 3 replicates). A series of hybridization signal intensity thresholds were used to mask probes with signals below the threshold (see Methods for details). For a particular probe (12 data points), all data points were kept if three or more probes were above the signal intensity threshold. Otherwise, all 12 probes were masked.

**Figure 2:**
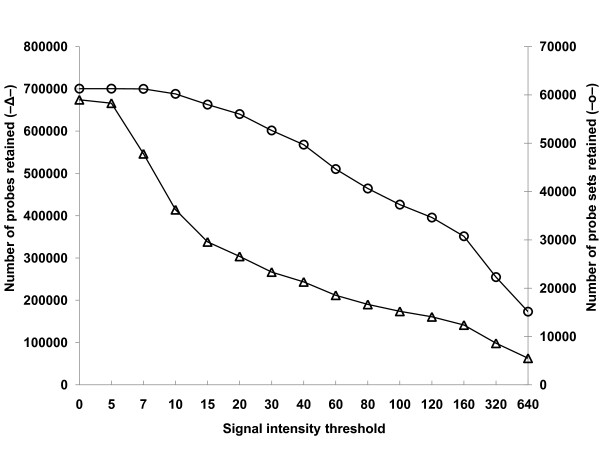
**The number of probes (―Δ―) and probe sets (―O―) retained after masking probes with a series of signal intensity thresholds**.

The gene (probe set) expression levels were derived from the signal intensities of the retained probes in each probe set by using Robust Multi-array Average (RMA) which involves background corrections, multi-chip quantile normalization and multi-chip summarization processes [33]. As expected, the number of probes retained decreased rapidly (from ~674,000 to ~62,000) while the number of probe sets retained decreased gradually (from ~61,000 to ~15,000) as signal intensity threshold increased (Figure 2).

**Figure 3:**
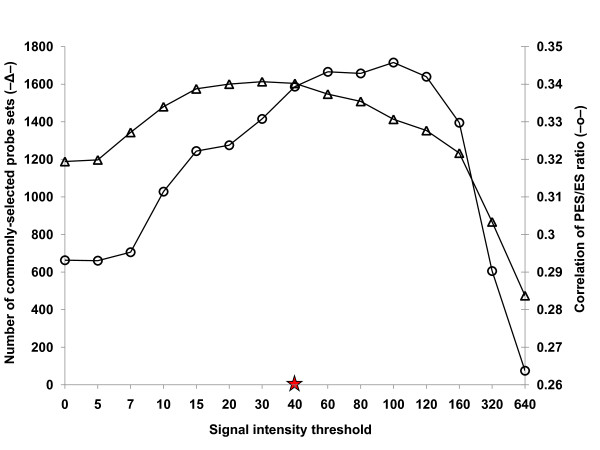
**Effect of masking probes over a range of signal intensity thresholds (0-640) on the number of probe sets commonly-selected in *M. truncatula *and alfalfa (―Δ―) and the correlation of the PES/ES hybridization intensity ratio for the commonly-selected genes (―O―). **A signal intensity threshold of 40 (red star) was selected to mask biased probes. Commonly-selected genes are defined as genes exhibiting at least a 2-fold difference in hybridization intensity expression ratio between PES and ES internodes (PES vs. ES, ≥ 2-fold difference) of both alfalfa and *M. truncatula*.

**Figure 4:**
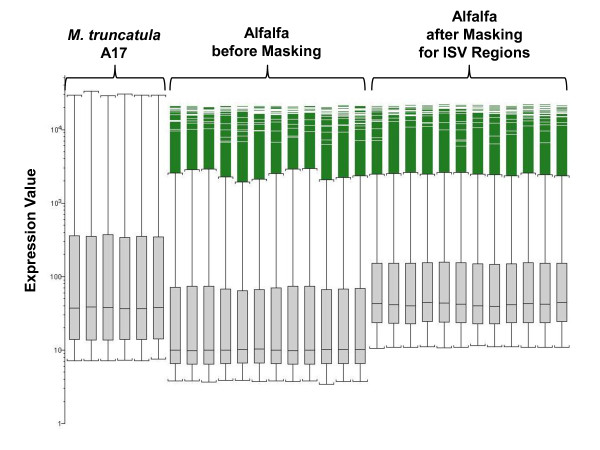
Box plots of 6 GeneChip data sets (two tissue types × 3 replications) from *M. truncatula *A17 genotype and 12 GeneChip datasets from alfalfa (2 genotypes × 2 tissue types × 3 replications) after masking for ISV regions (signal intensity threshold = 40)

In previous work, we used the *Medicago *GeneChip to compare gene expression in ES versus PES internodes of *M. truncatula *genotype A17 from which most of the probes on the GeneChip were designed [12]. The masking protocol developed in this study is based on the assumption that the ratio of expression of genes in ES and PES internodes (ES/PES) of alfalfa is very similar to that measured in these tissues in *M. truncatula *(A17). Using the *M. truncatula *dataset, we identified genes (probe sets) that exhibited at least a 2-fold difference in gene expression between ES and PES tissues. Next, we identified genes that exhibited at least a 2-fold difference in expression ratio between ES and PES internodes of alfalfa genotype 252 as probe signal intensity threshold was increased from 0 to 640. Genes exhibiting at least a 2-fold difference in expression ratio between ES and PES internodes of both alfalfa and *M. truncatula *are referred to as commonly-selected genes. The number of commonly-selected genes increased from ~1,200 to ~1,600 as the signal intensity threshold increased to a value of 40 reflecting increased detection sensitivity (Figure 3). The effect of masking biased probes with low signal intensity on the condensed signal intensity of the corresponding probe set is shown in Figure 4. Boxplots of the twelve GeneChip datasets (2 tissues × 2 species × 3 replicates) for alfalfa before and after masking ISV regions (probes with a signal intensity less than 40) show that masking increased mean signal intensity of the GeneChip dataset by approximately four-fold. The mean signal intensity after masking ISV regions is very close to the mean signal intensity of GeneChip dataset for stem internodes of *M. truncatula *(genotype A17). Overall, masking ISV regions increased the signal intensity of the *Medicago *probe sets which in turn increased the sensitivity of detecting alfalfa gene expression on the *Medicago *GeneChip.

To evaluate the effect of masking for ISV regions on the accuracy of measuring fold-changes in gene expression between PES and ES internodes of alfalfa, we examined the correlation of the hybridization intensity signal ratio of PES and ES internodes for the commonly-selected genes from the two *Medicago *species as signal intensity threshold was increased. The Pearson correlation coefficient of the PES/ES ratio between *M. truncatula *and alfalfa increased from 0.29 to 0.34 as signal intensity threshold increased up to about 100 reflecting increased accuracy (Figure 3). The decline in correlation detected at signal intensity thresholds above 100 may be due to masking too many informative probes. Although the highest correlation of the PES/ES ratio between the two *Medicago *species was achieved with a signal intensity threshold of 100, the number of commonly-selected genes was reduced (Figure 3). The data in Figure 3 show that the effect of masking on accuracy and sensitivity intersect at a signal intensity threshold of 40 where over 50,000 probe sets (about 85% of the total number on the Genechip) were retained (Figure 2). On the basis of these results, we used a signal intensity threshold of 40 for masking biased probes due to ISV regions. The use of this masking threshold significantly improved sensitivity (the number of expressed genes detected) while maintaining a high level of accuracy in measuring fold-difference in gene expression.

Most CSH studies in plants have used a genomic DNA-based strategy for probe selection [19,27,30]. To our knowledge, this study is the first to employ an RNA-based probe selection protocol to mask ISV regions when using a cross-species platform for transcript profiling in plants. The masking protocol that we developed has some advantages over previously reported masking protocols especially for crops with limited sequence information. For example, neither DNA hybridization [18] nor prerequisite sequence information [26] is needed to identify inter-species conserved probe sets. In addition, with careful experimental design including adequate replication, the masking protocol developed in this study is relatively simple to implement (see Methods). The protocol is based on the assumption that the ratio of gene expression in PES and ES internodes of two closely-related *Medicago *species (*M. truncatula *and *M. sativa*) is similar. In mammals, a similar approach involving comparisons of gene expression between organs has been used successfully in CSH studies [21]. In our study, the ratio of expression of probe sets in ES and PES internodes of *M. truncatula *was used to optimize both the sensitivity and the accuracy of detecting genes differentially expressed in alfalfa stem internodes. Our results suggest that a similar RNA-based approach for masking ISV regions could be successfully applied to other closely-related plant species where a microarray platform is available for one species.

Although the masking protocol used in this study is a useful tool for optimizing CSH GeneChip date, it does not correct for all bias in the data. It is important that candidate genes selected for further study based on masking results be validated by real-time quantitative RT-PCR. In addition, one limitation of a masking protocol based on RNA hybridization intensity is bias toward abundant transcripts. Low abundance genes (probes sets) would most likely be masked using this protocol.

### Masking probes for both ISV regions and SFPs

Single-feature polymorphisms (SFPs) are polymorphisms detected by single probes in microarrays [34]. Previously, we identified 10,890 SFPs between alfalfa genotypes 252 and 1283 using the GeneChip expression data files for ES and PES internodes [10]. These allelic variations between the two genotypes can bias transcript profiling by causing both false positives and false negatives [35,36]. The effect of masking for both ISV regions and SFPs (i.e. double-masking) on the number of probe sets retained was minimal. Only about 450 additional probe sets were lost after further masking for SFPs (data not shown). By masking for probes targeting both inter- and intra-species variable regions, we improved the quality of the CSH GeneChip data for the two alfalfa genotypes examined. The double-masking strategy employed in this study can be applied to other species when using a cross-species platform for transcript profiling between two genotypes.

### Effect of masking on detection of differentially expressed genes

**Figure 5:**
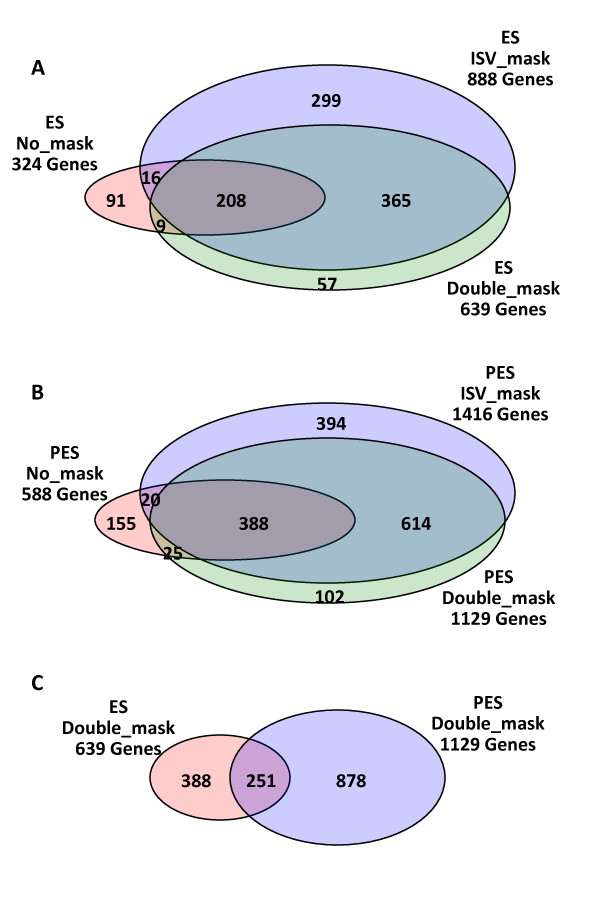
**Venn diagrams of the numbers of overlapping and non-overlapping genes differentially expressed between alfalfa genotypes 252 and 1283 in ES internodes (A) and PES internodes (B) before masking, after masking for ISV regions (threshold = 40), and after double-masking for ISV regions and SFPs, and (C) The number of overlapping and non-overlapping genes differentially expressed in ES and PES internodes of the two genotypes after double-masking. **A total of 52,911 probe sets (Mtr and Msa probe sets only) out of the 61,103 probe sets in the GeneChip^® ^Medicago Genome Array were used for analysis.

We used a t-test (p-value and FDR cutoff of 0.001 and 0.05, respectively) combined with an additional cut-off ratio of 2-fold to identify genes that were differentially expressed in ES and PES internodes of genotypes 252 and 1283 (Additional file 1). The Venn diagrams in Figure 5 show the number of differentially expressed genes identified before masking, after masking for ISV regions, and after double-masking (i.e. masking for both ISV regions and SFPs). Masking for ISV regions using a signal intensity threshold of 40 increased the number of differentially-expressed genes identified in ES and PES internodes by about 2.5-fold. After double-masking, the number of differentially expressed genes detected was decreased compared to the number detected after masking for ISV regions only (Figures 5A and 5B). This decrease is probably due to removal of probes associated with SFPs that were responsible for generating false positives for differentially expressed genes. After double-masking, 639 and 1129 genes were differentially expressed between the two alfalfa genotypes in ES and PES internodes, respectively (Figure 5C). A total of 251 genes were detected in both ES and PES internodes. From these results, we can estimate the number of putative false positives and false negatives that might have been caused by sequence variation (Figures 5A and 5B). For example in Figure 5A, 91 genes identified as being differentially expressed prior to masking, but not included after double-masking may be putative false positives caused by ISV regions and/or SFPs; 299 genes selected after masking for ISV regions but not included after double-masking may be putative false positives caused by SFPs; and 57 differentially expressed genes detected only after double-masking may be putative false negatives caused by ISV regions and/or SFPs (Figure 5A).

### Differences in gene expression between stem internodes of genotypes 252 and 1283

**Figure 6:**
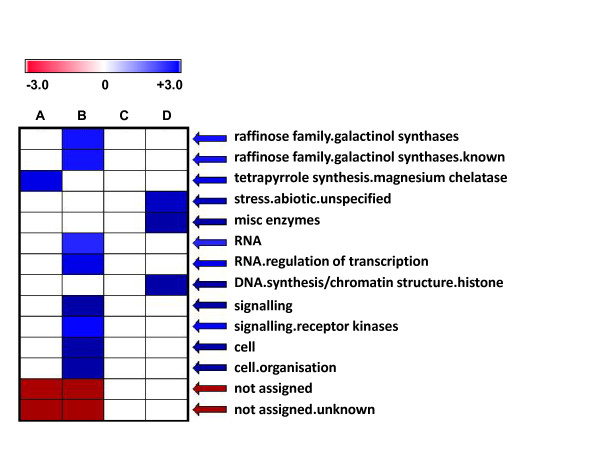
**Over-representation analysis of gene functional classes that are differentially expressed in ES and PES internodes of alfalfa genotypes 252 and 1283**. Columns: A, Genes up-regulated in 252 ES; B, Genes up-regulated in 252 PES; C, Genes down-regulated in 252 ES; D, Genes down-regulated in 252 PES compared to 1283 ES or PES. Blue and red indicate over- and under-representation of the corresponding class, respectively. The MapMan gene functional classification system consists of 34 major classes and their sub-classes [37]. The major classes (e.g., RNA) and their sub-classes (e.g., regulation of transcription) are separated by a "period" in the figure. The *z*-values for the functional class over- or under-represented in each group are provided in Additional file 2.

Genes that were differentially expressed in the internodes of alfalfa genotypes 252 and 1283 after double-masking were functionally classified using the MapMan gene functional classification system [10,37] (Additional file 1). To obtain an overview of gene functional classes that were differentially expressed in the two alfalfa genotypes, we conducted over-representation analysis using PageMan, a software tool for comparative analysis of gene ontology [38] (see Methods for details). Compared to all probe sets on the *Medicago *GeneChip, "regulation of transcription" and "signalling" classes were significantly over-represented among genes up-regulated in PES internodes of genotype 252 compared to genotype 1283 (Figure 6, Additional file 2).

**Figure 7:**
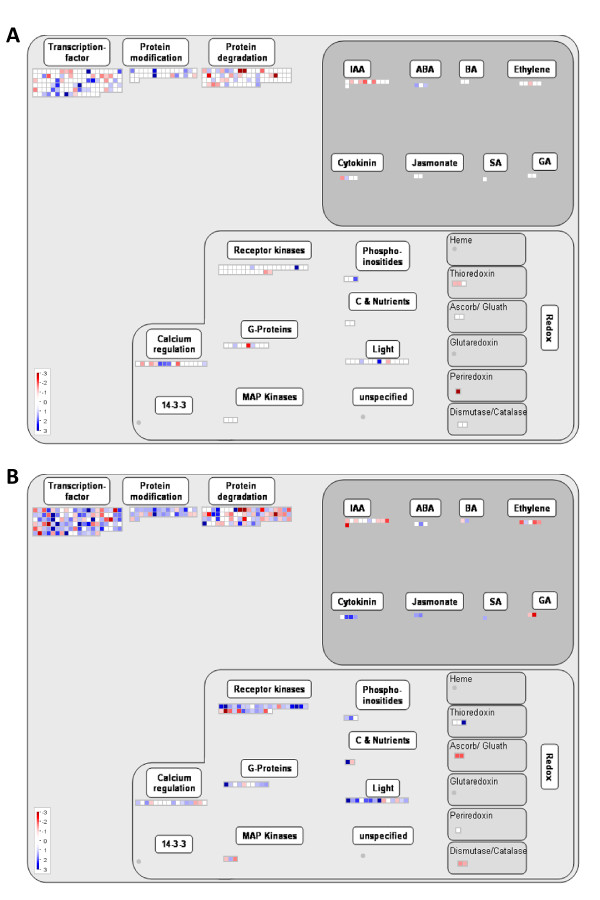
**MapMan overview showing regulatory genes that are differentially expressed in alfalfa genotypes 252 and 1283 in ES (A) and PES (B) internodes. Individual genes are represented by small squares. **The Log_2_(252/1283) values for the differentially expressed genes (p < 0.001, FDR < 0.05, ≥ 2-fold difference) were false color coded using a scale of -3 to +3. The intensity of blue and red colors indicates the degree of preferential expression of the corresponding genes in 252 and 1283, respectively. Color saturates at ±3 (8-fold difference or higher). See Methods for details. A complete list of the differentially expressed genes, corresponding MapMan functional categories, signal intensities and log ratios are provided in Additional file 1.

The transcript profiles of regulatory genes that were differentially expressed in ES and PES internodes of alfalfa genotypes 252 and 1283 are visually displayed in Figures 7A and 7B, respectively. A total of 115 putative transcription factor and 32 receptor kinase genes were differentially regulated between stems of the two alfalfa genotypes. The number of regulatory genes that were differentially expressed between the two genotypes was greater in PES internodes compared to ES internodes. The majority of putative transcription factor and receptor kinase genes that were up-regulated in PES internodes were found in genotype 252 (Figure 7, Additional file 1).

The role of various transcription factors in regulating cell wall development has been examined primarily in *Arabidopsis *and poplar (*Populus *spp.) [39]. For example, over-expression of some NAC (NAM/ATAF/CUC) and MYB proteins in *Arabidopsis *led to abnormal ectopic deposition of secondary cell walls and suppression of their functions resulted in a decrease in secondary cell wall thickening [40-47]. Some MYB family transcription factors also regulate the expression of genes involved in lignin biosynthesis [47-50]. Interestingly, a recent study suggested that the NAC family transcription factor *SND1 *(*SECONDARY WALL-ASSOCIATED NAC DOMAIN PROTEIN1*) acts as a master transcriptional switch for activating secondary cell wall biosynthetic pathways by regulating the expression of 11 transcription factors (1 homeobox-, 2 NAC- and 8 MYB-domain containing genes) essential for normal secondary cell wall development [45]. In the alfalfa genotypes examined in this study, putative NAC genes Mtr.50934.1.S1_at and Mtr.25921.1.S1_at were up-regulated in both ES and PES internodes of genotype 252 compared to the same tissues in genotype 1283. Three MYB genes (Mtr.6897.1.S1_at, Mtr.42648.1.S1_at, Mtr.44850.1.S1_at) were up-regulated in 252 PES internodes compared to 1283 PES internodes. Among these, Mtr.42648.1.S1_at is a putative homolog of *AtMYB63 *(86% identical at the protein level), a *SND-1 *regulated MYB transcription factor that specifically activates lignin biosynthetic genes during secondary cell wall formation in *Arabidopsis *[47]. *AtMYB63 *was specifically expressed in fibers and vessels undergoing secondary cell wall thickening. Over-expression of *AtMYB63 *resulted in specific activation of lignin biosynthetic genes causing ectopic deposition of lignin in normally non-lignifying cells. Suppression of *AtMYB63 *led to a reduction in secondary cell wall thickening and lignin content [47]. Mtr.42648.1.S1_at also has high sequence homology with two other *SND1*-regulated AtMYB genes (*AtMYB85 *and *AtMYB103*) with 73% and 70% identity at the protein level, respectively. Over-expression of *AtMYB103 *and *AtMYB85 *led to an increase in secondary cell wall thickening in fibers and ectopic deposition of lignin in epidermal and cortical cells in stems [45]. Dominant repression of *AtMYB103 *and *AtMYB85 *resulted in significantly reduced secondary cell wall thickening in fiber cells. We also identified numerous other differentially expressed transcription factor families that have not been previously reported to play a role in cell wall development. For example, zinc finger (14 genes total) and WRKY (18 genes total) were the most abundant families among the differentially expressed transcription factors in genotypes 252 and 1283. Other significant transcription factor families identified include bHLH, b-ZIP, and AP2/EREBP (Additional file 1).

Receptor-like kinases (RLKs) were also significantly over-represented among genes up-regulated in genotype 252 PES internodes compared to genotype 1283 PES internodes (Figure 7, Additional file 2). RLKs are known to play significant roles in plant growth, development and defence responses [51-53]. There are more than 600 RLKs in the *Arabidopsis *genome. Several recent reports suggested a significant role for RLKs in regulating cell wall development. For example, a loss of function mutant of *THESEUS1*, a plasma membrane receptor kinase, suppressed the ectopic lignification and growth inhibition phenotype of *prc1-1*, a recessive *CELLULOSE SYNTHASE 6 Arabidopsis *mutant, by repressing the induction of stress responses. These results suggested that the *THESEUS1 *RLK acts as sensor of cell wall integrity [54]. Mutations in two leucine-rich repeat (LRR) RLKs (*FEI1 *and *FEI2*) disrupt anisotropic expansion and the synthesis of cell wall polymers including cellulose biosynthesis [55]. WAKs (wall-associated Ser/Thr receptor kinases) are tightly bound to the cell wall and are thought to play a significant role in regulating cell wall function as well [56,57]. Among the 32 putative RLKs identified in genotypes 252 and 1283, 23 were up-regulated in 252 PES internodes, one was up-regulated in both ES and PES internodes of 252, and one was up-regulated in 252 ES internodes compared to 1283 ES or PES internodes. One of the RLKs up-regulated in 252 PES internodes (Mtr.9325.1.S1_at) is a homolog of *Arabidopsis FEI1*. Two putative WAKs (Mtr.13054.1.S1_at and Mtr.3807.1.S1_at) were up-regulated in 252 PES internodes as well.

**Figure 8:**
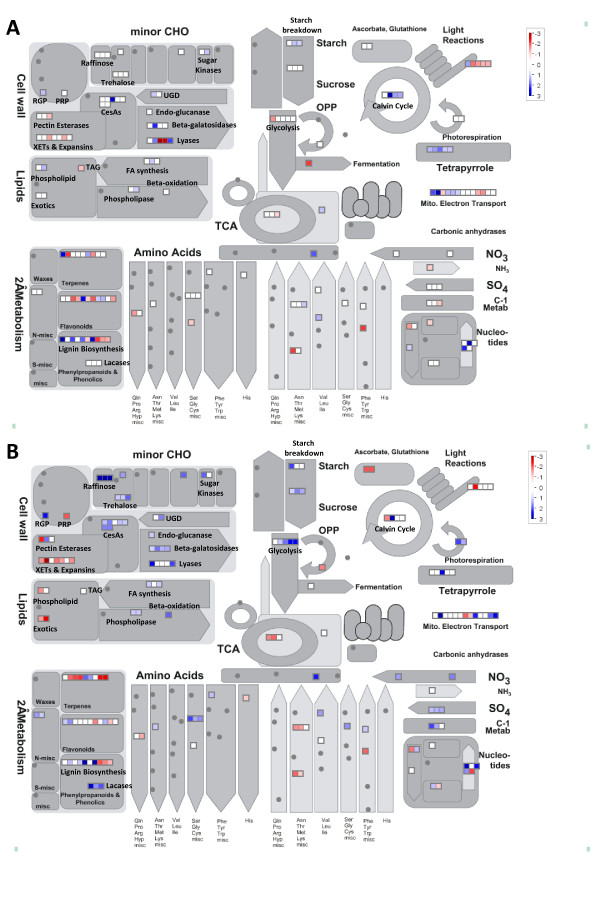
**MapMan overview of metabolism showing genes that are differentially expressed between alfalfa genotypes 252 and 1283 in ES (A) and PES (B) internodes. **The Log_2_(252/1283) values for the differentially expressed genes (p < 0.001, FDR < 0.05, ≥ 2-fold difference) were false color coded using a scale of -3 to +3. The intensity of blue and red colors indicates the degree of preferential expression of the corresponding genes in 252 and 1283, respectively. Color saturates at ±3 (8-fold difference or higher). See Methods for details. A complete list of the differentially expressed genes, corresponding MapMan functional categories, signal intensities and log ratios are provided in Additional file 1.

MapMan [37] was also used to provide an overview of metabolism genes that were differentially expressed in ES and PES internodes of genotypes 252 and 1283. Examples of metabolism-related genes that are differentially expressed between genotypes 252 and 1283 in ES and PES internodes are shown in Figures 8A and 8B (Additional file 1). A number of cell wall-related genes were differentially expressed in the two genotypes with the greatest number detected in PES internodes. For example, seven putative cellulose synthase (CesA) or CesA-like genes were differentially regulated between the two alfalfa genotypes. Six of these putative CesA or CesA-like genes (Mtr.5274.1.S1_s_at, Mtr.5242.1.S1_at, Mtr.13202.1.S1_at, Mtr.45005.1.S1_at, Mtr.10447.1.S1_s_at, and Mtr.5170.1.S1_at) were up-regulated in 252 PES internodes compared to 1283 PES internodes. Only one CesA gene (Mtr.28768.1.S1_at) was up-regulated in genotype 252 ES internodes compared to genotype 1283 ES internodes. In *Arabidopsis*, three CESA genes (*CesA4/IRREGULAR XYLEM5 [IRX5], CesA7/IRX3*, and *CesA8/IRX1*) are known to be involved in secondary cell wall biosynthesis [58-61]. Mtr.5242.1.S1_at and Mtr.13202.1.S1_at probe sets are putative homologs of CesA8. Mtr.5274.1.S1_s_at and Mtr.28768.1.S1_at are putative homologs of CesA4 and CesA7, respectively. Mtr.45005.1.S1_at and Mtr.5170.1.S1_at are the putative homologs of Arabidopsis *CSLD3 *(CELLULOSE SYNTHASE-LIKE D3) and *CSLD4*. *CSDLs *are thought to be involved in the synthesis of xylan, non-crystalline cellulose, and the galactan side chains of pectin or arabinogalactan proteins [62]. Other interesting cell wall genes that were up-regulated in genotype 252 PES internodes include UDP-glucose 6-dehydrogenase (UGD: Msa.2895.1.S1_at) and sucrose synthase (SuSy: Msa.2902.1.S1_at). Both genes are involved in the metabolism of UDP-D-glucose, a precursor for the synthesis of cellulose and cell wall matrix polysaccharides.

We also identified differentially expressed genes involved in lignin biosynthesis in the stems of alfalfa genotypes 252 and 1283 (Figure 8). For example, hydroxycinnamoyltransferase (*HCT*: Mtr.34427.1.S1_at) and caffeoyl-CoA 3-O-methyltransferase (*CCoAOMT*: Mtr.40942.1.S1_at) were up-regulated in both ES and PES internodes of genotype 252 compared to 1283. However, the degree of up-regulation in 252 was greater in PES internodes compared to ES internodes. In addition, three putative laccase genes (*LAC17*: Mtr.39737.1.S1_at, Mtr.45364.1.S1_at, and Mtr.4126.1.S1_at) and a putative 4-coumarate-CoA ligase gene (*4CL*: Mtr.42330.1.S1_at) were up-regulated in genotype 252 PES compared to genotype 1283. Interestingly, some genes involved in lignin biosynthesis were down-regulated in genotype 252 compared to genotype 1283. For example, two putative *CCoAOMT *genes (Mtr.31539.1.S1_at and Mtr.4850.1.S1_at) and cinnamyl-alcohol dehydrogenase (*CAD*: Mtr.8985.1.S1_at and Mtr.6181.1.S1_at) were down-regulated in genotype 252 compared to genotype 1283.

### Differences in gene expression between stem internodes of genotypes 252 and 1283 are consistent with differences in cell wall composition

Overall, our results show significant up-regulation of a number of regulatory and cell wall-related genes in PES internodes of genotype 252 compared to genotype 1283. Many of the regulatory genes that were up-regulated are putative transcription factors and receptor kinases. Several of these up-regulated genes are known to play a role in the development of secondary xylem in *Arabidopsis *(e.g., *AtMYB63*, *AtMYB85*, *AtMYB103*, and *FEI1*). In addition, putative CesA genes that play a role in secondary cell wall development and putative genes involved in lignin synthesis were up-regulated in 252 PES internodes compared to 1283 PES internodes. The up-regulation of these regulatory and cell wall-related genes may play a role in the greater cell wall concentration and modified composition of PES internodes of genotype 252. On a dry matter basis, cellulose and lignin concentrations of stems of flowering plants (primarily PES internodes) are significantly higher in genotype 252 compared to genotype 1283 (302 g kg^-1 ^vs. 257 g kg^-1 ^for cellulose and 117 g kg^-1 ^vs. 98 g kg^-1^for Klason lignin, respectively) [10]. The greater cellulose and lignin concentrations in stems of genotype 252 compared to genotype1282 are associated with an 11% increase in total cell wall dry matter and a reduction in concentration of pectin sugar residues in the cell wall. The genotypic differences in cell wall concentration and composition of PES internodes are consistent with greater deposition of secondary xylem in internodes of genotype 252 compared to genotype 1283. Previous research has shown that increased development of secondary xylem increases cell wall concentration in alfalfa stems expressed on a dry weight basis [63]. Furthermore, the thick secondary walls of this tissue are rich in cellulose, xylan and lignin, but contain little if any pectin compared to primary cell walls [63]. The candidate genes identified in this study, especially transcription factor genes and genes involved in secondary cell wall synthesis, may play important roles in the development of secondary xylem in PES internodes of alfalfa. Future research involving transgenic approaches will be used to evaluate the role of these genes in the deposition of secondary xylem in alfalfa stems. Modifying the amount and composition of secondary xylem in stems of alfalfa will improve the value of alfalfa as a cellulosic feedstock.

### Validation of selected candidate genes

A subset of 50 differentially expressed candidate genes from three functional categories (regulatory, signalling and cell wall-related genes) was initially selected for real-time quantitative RT-PCR validation. However, only 34 of these genes produced a single amplicon based on dissociation curves (see Methods for details). Most primers for real-time quantitative RT-PCR were designed using *M. truncatula *sequences because most probe sets selected for validation were designed from *M. truncatula *sequences. Sequence variation between the two *Medicago *species and among multi-gene families within species may explain the lower than expected RT-PCR success rate.

A total of 13, 15, and 6 candidate genes from cell wall, transcription factor, and signal transduction categories, respectively, were used for real-time quantitative RT-PCR validation (Table 1). The GeneChip data with no masking indicated that 12 of the 34 candidate gene examined were differentially expressed between the two alfalfa genotypes. The quantitative RT-PCR validation data (2 plates × 3 wells × 2 genotypes =12 data points) revealed that of the 12 probes sets detected with no masking, 10 (83.3%) were true positives (Table 1). Two probe sets (16.7%) were identified as false positives. After double-masking the GeneChip data, an additional 22 genes were identified as differentially expressed (Table 1). The quantitative RT-PCR validation data for the 22 genes that were identified only after double-masking indicated that 19 (86.4%) were true positives. Three probe sets (13.6%) were identified as false positives. Overall, these results indicate a significant increase in detection power after double-masking (about a 2.8-fold increase in sensitivity) with little change in the false positive rate.

**Table 1:** Candidate genes used for real-time quantitative RT-PCR validation.

		**t-test**^1^		**Ratio**^2^		
			
Annotation	Probe Set ID	No mask	Double- mask	No mask	Double- mask	ΔΔC_T_^3^
**Cell Wall-related Genes**						
Chitinase	Msa.3005.1.S1_at	+	+	-2.8	-3.4	-2.2**
Peroxidase	Mtr.40028.1.S1_at	-	+	-4.5	-5.2	-2.2**
Chitinase	Mtr.40130.1.S1_at	+	+	-1.8	-2.1	-1.2**
Reversibly glycosylated polypeptide	Mtr.4595.1.S1_at	-	+	0.8	2.4	1.9**
Cellulose synthase-like	Mtr.45005.1.S1_at	-	+	0.8	1.1	0.3**
Endo-1,4-beta glucanase	Mtr.31102.1.S1_at	-	+	0.9	1.1	1.1**
Xyloglucan endotransglusoxylase	Mtr.6499.1.S1_at	-	+	-0.7	-1.6	-1.3**
UDP-glucose dehydrogenase	Msa.2895.1.S1_at	+	+	1.3	1.6	0.4*
Acetyl-CoA C-acetyltransferase	Mtr.10429.1.S1_at	-	+	-0.5	-2.7	-2.3**
Cinnamyl-alcohol dehydrogenase	Mtr.8985.1.S1_at	-	+	-0.3	-1.3	-0.9**
Caffeoyl-CoA O-methyltransferase	Mtr.4850.1.S1_at	-	+	-1.3	-2	-1.5**
Glycosyl hydrolase	Mtr.2288.1.S1_at	-	+	0.4	2	0.6
UDP-glucosyl transferase	Mtr.37847.1.S1_at	+	+	2.2	4.1	3.0**
**Transcription Factors**						
LOB gene family	Mtr.28784.1.S1_at	-	+	1.8	2.4	1.2*
bZIP transcription factor	Mtr.17513.1.S1_s_at	+	+	2.3	2.8	2.7**
C2H2 zinc finger family	Mtr.972.1.S1_at	-	+	2	3.2	2.6**
C2H2 zinc finger family	Mtr.50483.1.S1_at	+	+	1.6	1.5	1.0**
C2H2 zinc finger family	Mtr.39849.1.S1_at	-	+	-1.5	-2.8	-1.4**
Homeobox transcription factor	Mtr.24122.1.S1_at	+	+	-1	-1.3	-0.4
Homeobox transcription factor	Mtr.46916.1.S1_at	-	+	-0.9	-1.9	-1.1**
Homeobox transcription factor	Mtr.38132.1.S1_a_at	-	+	-0.6	-1	-0.7**
JUMONJI family	Mtr.17177.1.S1_at	-	+	2	2.1	3.0**
MYB transcription factor	Mtr.42648.1.S1_at	-	+	0.6	2.4	1.0**
NAC transcription factor	Mtr.14808.1.S1_at	-	+	-0.9	-1.2	-0.7*
PHD finger family	Mtr.39174.1.S1_at	-	+	-1.1	-1.9	-0.2
Zinc finger family	Mtr.33527.1.S1_at	+	+	3.2	3.6	2.7**
WRKY family	Mtr.23120.1.S1_s_at	+	+	3.8	3.4	3.8**
WRKY family	Mtr.12866.1.S1_at	-	+	2.1	2.2	2.2**
**Signal Transduction Genes**						
Transducin family	Mtr.11654.1.S1_at	-	+	0.8	2.1	1.8
FAR1-related	Mtr.47127.1.S1_at	+	+	3	3	2.1**
MAP kinase	Mtr.5102.1.S1_at	+	+	-1.3	-1.7	-0.8**
Receptor-like kinase	Mtr.1545.1.S1_at	-	+	0.7	2.5	4.2**
Receptor-like kinase	Mtr.9480.1.S1_at	+	+	1.3	1.5	0.4
Receptor-like kinase	Mtr.3024.1.S1_at	-	+	0.6	2.1	2.2**

^1^+ indicates genes that were detected as differentially-expressed between PES internodes of genotypes 252 and 1283 after a t-test (p < 0.001, FDR < 0.05, ≥2-fold difference) using the GeneChip data with no masking or after double-masking.

^2^Log_2 _(252PES/1283PES) value obtained from the GeneChip data with no masking or after double-masking.

^3^ΔΔC_T _= [C_T_(1283PES) - C_T_(18s)] — [C_T_(252PES) - C_T_(18s)] from real-time quantitative RT-PCR data. C_T_18s rRNA values were stable in the tissues examined and were used to normalize data. *,** indicates probe sets that were detected as differentially expressed after a t-test (*P *< 0.01 or P < 0.001) respectively, The t-test of the real-time quantitative RT-PCR data for PES internodes of genotypes 252 and 1283 was performed with ΔC_T _values (12 data points = 2 plates × 3 wells × 2 genotypes).

**Figure 9:**
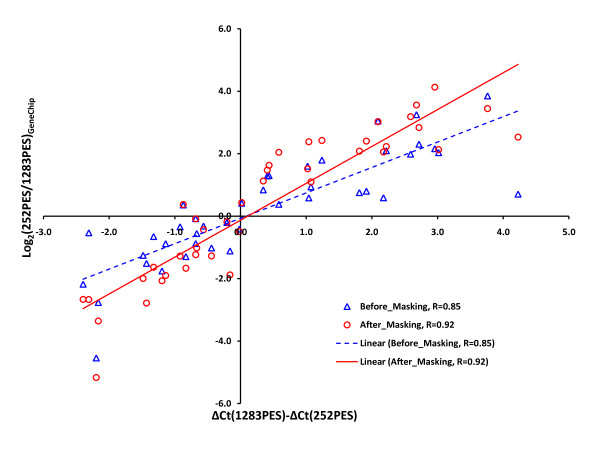
**ΔΔC_T _values obtained from the real-time quantitative RT-PCR data for the selected candidate genes (Table 1) plotted against log_2_(252PES/1283PES) hybridization intensity ratio values from the GeneChip data before (blue triangles) and after (red circles) masking**.

Next, we examined the effect of double-masking on the accuracy of measuring fold-differences in gene expression by plotted ΔΔC_T _values obtained from the real-time quantitative RT-PCR data against log_2_(252PES/1283PES) values from the GeneChip data before and after masking (Figure 9). The results show a linear relationship between the ΔΔC_T _value from real-time quantitative RT-PCR and the log ratio both before and after masking. Masking increased the *Pearson *correlation coefficient (*R*) from 0.85 to 0.92 (Figure 9). The stronger positive correlation between the two data sets after double-masking indicates increased accuracy.

### Physical mapping of differentially expressed genes

**Figure 10:**
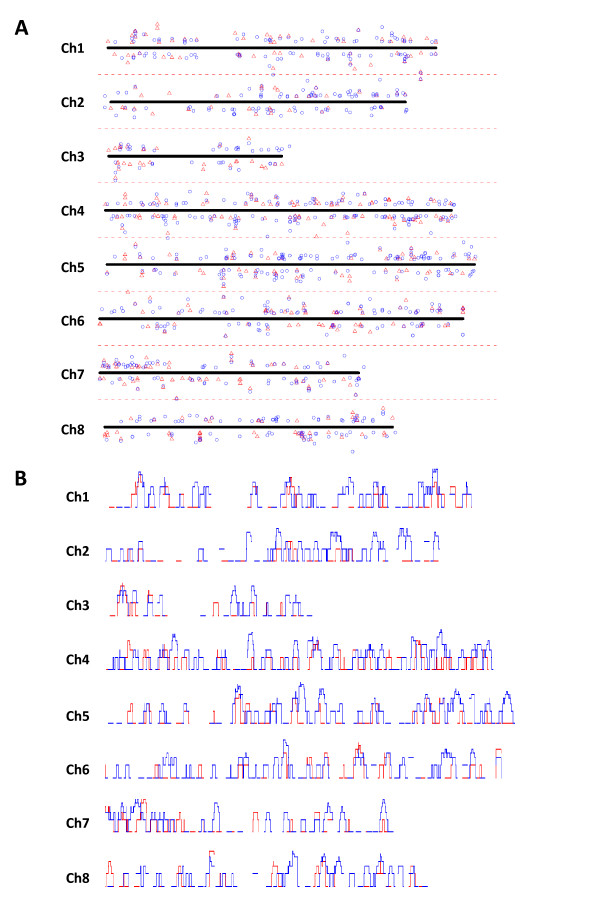
**(A) Physical mapping of differentially expressed genes (p < 0.001, FDR < 0.05, ≥ 2-fold difference) between alfalfa genotypes 252 and 1283 in ES internodes (red triangles) and PES internodes (blue circles) onto putative orthologous loci in the *Medicago truncatula *chromosomes 1 through 8 and (B) Comparison of the frequency distribution on *Medicago *chromosomes 1 through 8 for the genes differentially expressed between alfalfa genotypes 252 and 1283 in ES (red line) and PES (blue line) internodes**. **(A)**The corresponding physical loci for genes are indicated on the solid line (x-axis) for each chromosome. The y- axis, indicated within the doted lines above and below the solid line, provides a measure of the Log_2_(252/1283) value for each gene [(Log_2_(252/1283) = 0 for solid line (x-axis)]. The symbols located above and below the zero center line represent genes up- and down-regulated in 252 compared to 1283 in ES (red triangles) and PES (blue circles) internodes, respectively. The vertical distance of each symbol from the zero center line indicates the degree of differential gene expression [Log_2_(252/1283)] for the corresponding genes on each chromosome. The orthologous loci of the genes selected on *M. truncatula *chromosomes are provided in Additional file 3. **(B)**The *y*-axis for each panel represents relative gene frequency in a 50 kb sliding window that reads gene frequency over 10 kb intervals.

Alfalfa and *M. truncatula *share the same eight orthologous basic chromosome sets but with different ploidy levels; 2n = 4x = 32 for alfalfa and 2n = 2x = 16 for *M. truncatula*. In addition, previous studies reported a significant level of colinearity in gene content and order between diploid and tetraploid alfalfa [64] and also between diploid alfalfa and *M. truncatula *[65,66]. The *Medicago *genome sequencing project has released genome sequence version 2.0 (http://www.medicago.org/genome/) which contains about 220 Mbp of the total estimated 300 Mbp of *M. truncatula *euchromatin. A total of 38,844 non-overlapping coding sequences have been annotated to some extent with locus information to the corresponding chromosomes. To examine trends in the chromosomal distribution of genes that were differentially expressed between alfalfa genotypes 252 and 1283, we mapped 1044 differentially expressed genes (out of the total of 1624) onto their corresponding orthologous loci on *M. truncatula *chromosomes (Figure 10, Additional file 3). Of these mapped genes, 445 and 775 were differentially expressed in ES and PES internodes, respectively (Figure 10A). A total of 176 genes were differentially expressed in both ES and PES internodes of the two genotypes.

Although differentially expressed genes between alfalfa genotypes 252 and 1283 were distributed over all chromosomes, regions of high frequency were evident (Figure 10A). The frequency of differentially expressed genes in ES and PES internodes was analyzed in a 50 kb sliding window along chromosomes. The results indicated possible chromosomal clustering of genes with the same expression pattern (Figure 10B). For example, in several chromosomal segments, we observed possible clustering of genes that were up-regulated in 252 PES internodes compared to 1283 PES internodes (Figure 10B, chromosomal regions where only blue peaks were observed). These results suggested possible co-regulation of differentially expressed genes found in localized chromosomal regions (Figure 10). We used the simulation method described by Grant et al. [67] to statistically analyze whether the clustering patterns observed for differentially expressed genes with similar expression patterns (i.e. genes up-regulated in 252 PES internodes compared to 1283 PES internodes) were non-random (see Methods for details). The difference between experimental data (selected gene distribution) and simulated data (random distribution) was considered statistically significant if the absolute value of (experimental data — simulation mean)/(simulation standard deviation) was ≥ 2 (see Methods for details). The results indicated statistically significant clustering of two to four differentially expressed genes with a similar expression pattern within both 50 kb and 100 kb windows in the genome (Table 2, Additional file 4).

**Table 2:** Clustering simulation analysis of co-expressed genes (50 kb window) in ES and PES internodes of alfalfa genotypes 252 and 1283.

Gene Category*	Tissue Type	Number of Genes^†^	Simulation Mean^‡^	Simulation SD	Experimental Data^§^	SD from Simulation Mean^¶^
up in 252	ES	1.0	192.8	5.3	163.0	-5.6
		2.0	7.7	2.6	16.0	3.2
		3.0	1.0	0.4	3.0	5.6

down in 252	ES	1.0	212.4	5.9	189.0	-4.0
		2.0	9.2	2.8	13.0	1.3
		3.0	1.2	0.5	3.0	3.7

up in 252	PES	1.0	309.5	8.3	262.0	-5.7
		2.0	20.3	4.0	34.0	3.4
		3.0	1.7	0.9	4.0	2.6
		4.0	1.0	0.1	3.0	20.0

down in 252	PES	1.0	354.3	9.5	307.0	-5.0
		2.0	26.9	4.5	46.0	4.3
		3.0	2.1	1.2	5.0	2.4

*Genes up- or down-regulated in genotype 252 compared to genotype 1283.

^†^Number of genes identified within the 50 kb bin of the simulation.

^‡^Average number of bins with the corresponding gene numbers from replicated simulation (×2000).

^§^Number of 50 kb bins with the corresponding gene number from this study.

^¶^(Experimental Data - Simulation Mean)/(Simulation SD). Value of 2 or greater indicates statistically significant clustering of genes.

To some degree, the clustering of co-expressed genes detected in this study may represent tandem repeats of duplicated genes. During the physical mapping process, some probe sets were targeted to closely linked multiple loci on the *M. truncatula *genome. If multiple loci hits per probe set were detected, we mapped only the top hit locus for each differentially expressed probe set onto the *M. truncatula *genome. By doing so, we reduced the chances that clusters detected in our cluster simulation analysis were due to tandem repeats of duplicated genes. Thus, the majority of co-expressed gene loci used for clustering simulation analysis are sequence unrelated. Previous studies conducted in other model systems reported chromosomal clustering of co-expressed genes even after removing duplicated genes [68,69].

Numerous studies have reported co-expression of neighbouring genes in eukaryotes [70-74] including *Arabidopsis *[68,75,76] and rice [77]. Co-regulated gene clusters often share the same biological functions and/or are in the same pathway [72,75]. These co-expressed genes could be regulated by the same transcription factor [72] or share the same promoter elements [71] for co-regulation. Natural selection may promote the clustering of co-expressed genes as well [70]. However, the mechanism behind the clustering of co-expressed genes is still unclear.

Chromosomal segments with clusters of co-expressed candidate genes will be useful for alfalfa breeding, especially for wide-crosses involving introgression of foreign chromosomal segments from alien species into elite alfalfa cultivars. In addition, the genomic DNA sequence of multiple candidate genes can be obtained by sequencing a BAC containing the candidate gene cluster.

## Conclusion

Masking biased probes due to inter-species variable (ISV) regions and SFPs increased the sensitivity and accuracy of the transcript profiling data for alfalfa when using the *Medicago *GeneChip as a cross-species platform. The masking protocol developed in this study can be applied to other CSH studies involving the use of GeneChips for transcript profiling. The transcript profiling data, indicating up-regulation of putative cellulose and lignin genes involved in secondary cell wall thickening in 252 PES internodes compared to 1283 PES internodes, is consistent with difference in cell wall concentration and composition between the two genotypes. Numerous cell wall and regulatory genes that may contribute to differences in cell wall composition and concentration of the two alfalfa genotypes were identified. These candidate genes will be useful for improving alfalfa as a cellulosic feedstock via a transgenic approach. Physical mapping and clustering simulation analysis of the differentially expressed alfalfa genes on orthologous loci of *M. truncatula *suggested chromosomal regions where statistically significant co-expression of neighbouring genes occurred.

## Method

### Plant materials

Alfalfa [*Medicago sativa *(L) subsp. *sativa*] clonal lines 252 and 1283 were selected as previously described [10].

### RNA extraction, labelling and GeneChip hybridization

Elongating and post-elongation stem internodes of genotypes 252 and 1283 grown in the greenhouse were harvested as previously described [10]. Methods used for RNA extraction, labelling, and GeneChip hybridization were previously described [10]. The raw data cel files used in this study are available in the NCBI Gene Expression Omnibus (http://www.ncbi.nlm.nih.gov/geo/query/acc.cgi?acc=GSE13602).

### Masking probes targeting variable regions

First, we created a series of probe mask files based on normalized probe intensity signals. Briefly, all cel files were background corrected and quantile-normalized at the probe level using affy package from Bioconductor (http://bioconductor.org). Based on the minimums, maximums, and different quantile distributions of the probe signal intensities, we chose several intensity points (5, 7, 10, 15, 20, 30, 40, 60, 80, 100, 120, 160, 320, 640, 1280, and 2560) for masking. One mask file was created for each probe intensity point. If the intensity of a probe was lower than the masking intensity in a defined percentage of all cel files, the probe was masked for all samples. The defined percentage (P) was determined by the number of replicates in each sample type and the number of sample types:

where Ts is the total number of sample files, R is the replicate number of each sample type and S is the number of sample types. The *floor *and *ceiling *are mathematical functions that map a real number to the next smallest and next largest integer, respectively. In this study, we used genotypes 252 and 1283 with ES and PES internode tissues for each genotype (S = 4) and three replicates (R = 3) for a total of 12 sample files (Ts = 12, P = 0.83). Based on the defined percentage (P), we created a series of mask files at each intensity point for all samples. A file was also created for masking the probe locations of the previously identified 10,890 single-feature polymorphisms (SFPs) in ES and PES internodes of alfalfa genotypes 252 and 1283 [10].

Next, we applied the series of masking files to the cel files of ES and PES internodes of genotype 252 using Expressionist Refiner module (http://www.genedata.com). Briefly, the raw data cel files were loaded into Refiner with the masking files. The RMA algorithm summarized the probe-level signals into probe set expression indexes. The resulting expression index files were evaluated by comparing them to the expression files obtained from the same internode tissues in the reference species, *M. truncatula*. For each signal intensity, we examined the correlation of the PES/ES expression ratio for the commonly-selected genes from the two *Medicago *species. Commonly-selected genes are defined as genes that exhibit at least a 2-fold difference in gene expression in ES and PES internodes of the two species. The masked expression data that yielded the best performance, as evaluated by optimization of both sensitivity and accuracy, was selected for differential gene expression analysis.

### Detection of differentially expressed genes

After selection of the optimum intensity threshold for masking, the differentially expressed genes were identified in the masked data set by applying a t-test to the expression values in ES or PES internodes (3 replications for each tissue type) of the two alfalfa genotypes (e.g., 252 ES vs. 1283 ES) with a p-value and FDR cutoff of 0.001 and 0.05, respectively. An additional ratio cutoff of 2-fold was applied using the Genedata Expressionist Analyst module (http://www.genedata.com/). The gene expression signals corresponding to the bacterial microsymbiont *(Sinorhizobium meliloti*) probe sets were excluded in this analysis.

### Functional classification and over-representation analysis

The MapMan gene functional classification system [37] was assigned to the probe sets on the *Medicago *GeneChip following the method previously described [10]. The functional class over-representation analysis was performed using PageMan [38] as previously described [10] except that the log_2_(252/1283) values for ES and PES internodes were given to the selected probe sets and the remainder of the probe sets on the GeneChip (not selected) were given a false expression value of "zero". For over-representation analysis, the z-value cuttoff was set as 1 after Bonferroni correction.

### Real-time quantitative RT-PCR

Total RNA used for GeneChip hybridization was also used to make cDNAs for real-time quantitative RT-PCR. First strand cDNAs for each sample were made using random hexamers and Taqman Reverse Transcription Reagents (Applied Biosystems, CA) following the manufacturer's recommendations. Gene specific primers for the selected probe sets were designed based on the consensus sequences (http://www.affymetrix.com) using Primer Express (Applied Biosystems, CA) (Additional file 5). Samples and standards were run in triplicate on each plate and repeated on at least two plates using SYBR-Green PCR Master Mix (Applied Biosystems, CA) on a GeneAmp 7500 Sequence Detection System (Applied Biosystems, CA) following the manufacturer's recommendations. Real-time quantitative RT-PCR was performed in a 20 μl reaction containing 7 μl ddH_2_O, 10 μl 2× PCR mix, 1 μl forward primer (4 μM), 1 μl reverse primer (4 μM), and 1 μl of template cDNA (10 ng/μl). The PCR conditions were two minutes of pre-incubation at 50°C, 10 minutes of pre-denaturation at 94°C, 40 cycles of 15 seconds at 95°C and one minute at 60°C, followed by steps for dissociation curve generation (30 seconds at 95°C, 60 seconds at 60°C and 30 seconds at 95°C). The 7500 System SDS software v.1.2.2 was used for data collection and analysis. Dissociation curves for each amplicon were carefully examined to confirm the specificity of the primer pair used. Relative transcript levels for each sample were obtained using the "comparative C_T _method". The threshold cycle (C_T_) value obtained after each reaction was normalized to the C_T _value of 18S rRNA. The relative expression level was obtained by calibrating the ΔΔC_T _values for other samples using a normalized C_T _value (ΔΔC_T_) for the PES internodes of alfalfa genotype 252.

### Physical mapping and frequency distribution

The *Medicago *genome release version 2.0 (Mt2.0) (http://www.medicago.org/genome/) contains 38,844 coding sequences with various degrees of annotation and predicted chromosome locations. We used these coding sequences to search against the *Medicago *GeneChip Probe consensus sequences database  (http://www.affymetrix.com) using blastn [78] with match matrix BLOSUM62 and a mismatch penalty of -3. We chose the blast parameter E value (0.0001) and bit score (100) for hit cutoff. If there were multiple loci hits per probe set, only the top hit locus was mapped for each probe set to minimize the effect of tandem repeats during the clustering simulation analysis. This analysis generated putative orthologous chromosome locations for 36,709 of the *Medicago *GeneChip probe sets. We used an R script to map the differentially expressed genes (p < 0.001, >2-fold difference) between alfalfa genotypes 252 and 1283 [ ES internodes (red triangles) and PES internodes (blue circles)] onto the putative orthologous loci in the *M. truncatula *chromosomes 1 through 8.

Frequencies of differentially expressed genes on chromosomes 1 through 8 in ES and PES internodes were examined in a 50 kb sliding window. For each tissue, the physical location information of the differentially expressed genes on the *M. truncatula *chromosomes was extracted. The frequency of genes selected within a sliding 50 kb window was calculated. This sliding window shifted every 10 kb along the chromosome. Within each window, the calculated gene frequency was plotted against chromosome distance in kb.

### Clustering simulations

The simulation protocol described by Grant et al. [67] was used to test for clustering of selected differentially expressed candidate genes on chromosomes. Mt2.0_pseudomolecule contains a total 266,102,767 bases on 8 chromosomes. This genome sequence was partitioned into a total of 3,856 and 2,122 bins based on 50 kb and 100 kb windows, respectively. The simulation program randomly positioned a defined number of selected genes on the genome and the number of bins with different frequency of assigned genes was determined. The simulation was repeated 2,000 times. The difference between experimental data (selected gene distribution) and simulated data (random distribution) was considered statistically significant if the absolute value of (experimental data — simulation mean)/(simulation standard deviation) was ≥ 2. A significant difference is indicative of clustering within the defined 50 kb or 100 kb window.

## Abbreviations

ES: elongating stem; PES: post-elongation stem; SFP: single-feature polymorphism; CSH: cross-species hybridization; EST: expressed sequence tag; SNP: single nucleotide polymorphism; RMA: robust multi-array average; ISV: inter-species variable; RLK: receptor-like kinase.

## Authors' contributions

SY and WX performed the computational anlaysis involved in the electronic masking of probes, functional classification and over-representative analysis. SY conducted the real-time quantitative RT-PCR validation. MT performed the microarray work for the generation of GeneChip data. JL identified the genotypes used in the study. HJ conducted the cell wall analysis of the alfalfa genotypes. All authors contributed to the analysis of results and writing of the manuscript. All authors read and approved the final manuscript.

## Supplementary Material

Additional file 1 **Genes differentially expressed in ES and PES internodes of alfalfa genotypes 252 and 1283**. A table listing genes (probe sets) that were differentially expressed in ES and PES internodes of alfalfa genotypes 252 and 1283. Also included are corresponding p-values, FDR values, and MapMan functional class and description. Click here for file

Additional file 2 **Over-representation analysis of gene functional classes that are differentially expressed in ES and PES internodes of alfalfa genotypes 252 and 1283**. A table showing *z*-values for functional classes over-represented in Figure 6. Click here for file

Additional file 3 **Orthologous loci of the alfalfa candidate genes on *M. truncatula *chromosomes**. A table listing orthologous loci of the alfalfa candidate genes on *M. truncatula *chromosomes. Click here for file

Additional file 4 **Clustering simulation analysis of co-regulated genes**. A table showing the result of clustering simulation analysis of co-regulated genes with a 100 kb window. Click here for file

Additional file 5 **Primers used for real-time quantitative RT-PCR validation**. A table listing primers used for real-time quantitative RT-PCR validation of the candidate genes selected from three functional categories (see Table 1). Click here for file

## References

[B1] Michaud R Lehman WF Rumbaugh MD World distribution and historical development Alfalfa and alfalfa improvement -- Agronomy Monograph no. 29 1988 Madison, WI: ASA-CSSA-SSSA 25 91

[B2] Samac DA Jung H-JG Lamb JFS Minteer SD Development of alfalfa (*Medicago sativa *L.) as a feedstock for production of ethanol and other bioproducts Alcoholic Fuels 2006 Boca Raton, FL: CRC Press 79 98

[B3] National Agricultural Statistics Service On-line resource 2008 http://www.nass.usda.gov

[B4] Russelle MP Birr AS Large-scale assessment of symbiotic dinitrogen fixation by crops: Soybean and alfalfa in the Mississippi river basin Agron J 2004 96 1754 1760

[B5] Angers DA Changes in soil aggregation and organic carbon under corn and alfalfa Soil Sci Soc Am J 1992 56 1244 1249

[B6] Russelle MP Lamb JFS Turyk NB Shaw BH Pearson B Managing nitrogen contaminated soils: benefits of N_2_-fixing alfalfa Agron J 2007 99 738 746 10.2134/agronj2005.0325

[B7] Lamb JFS Jung H-JG Sheaffer CC Samac DA Alfalfa leaf protein and stem cell wall polysaccharide yields under hay and biomass management systems Crop Sci 2007 47 1407 1415 10.2135/cropsci2006.10.0665

[B8] Chapple C Ladisch M Meilan R Loosening lignin's grip on biofuel production Nature Biotech 2007 25 746 748 10.1038/nbt0707-746 17621299

[B9] Chen F Dixon RA Lignin modification improves fermentable sugar yields for biofuel production Nature Biotech 2007 25 759 761 10.1038/nbt1316 17572667

[B10] Yang SS Xu WW Tesfaye M Lamb JFS Jung H-JG Samac DA Vance CP Gronwald JW Single-feature polymorphism discovery in the transcriptome of tetraploid alfalfa Plant Genome 2009 2 224 232 10.3835/plantgenome2009.03.0014

[B11] Tesfaye M Silverstein KAT Bucciarelli B Samac DA Vance CP The Affymetrix *Medicago *GeneChip^® ^array is applicable for transcript analysis of alfalfa (*Medicago sativa*) Func Plant Biol 2006 33 783 788 10.1071/FP06065 32689289

[B12] Tesfaye M Yang SS Lamb JFS Jung H-JG Samac DA Vance CP Gronwald JW VandenBosch KA *Medicago truncatula *as a model for dicot cell wall development Bioenergy Res 2009 2 59 76 10.1007/s12155-009-9034-1

[B13] Enard W Khaitovich P Klose J Zöllner S Heissig F Giavalisco P Intra- and interspecific variation in primate gene expression patterns Science 2002 296 340 343 10.1126/science.1068996 11951044

[B14] Cáceres M Lachuer J Zapala MA Redmond JC Kudo L Geschwind DH Elevated gene expression levels distinguish human from non-human primate brains Proc Natl Acad Sci USA 2003 100 13030 13035 10.1073/pnas.2135499100 14557539 PMC240739

[B15] Horvath DP Schaffer R West M Wisman E *Arabidopsis *microarrays identify conserved and differentially expressed genes involved in shoot growth and development from distantly related plant species Plant J 2003 34 125 134 10.1046/j.1365-313X.2003.01706.x 12662315

[B16] Meiklejohn CD Parsch J Ranz JM Hartl DL Rapid evolution of male-biased gene expression in *Drosophila* Proc Natl Acad Sci USA 2003 100 9894 9899 10.1073/pnas.1630690100 12907700 PMC188344

[B17] Michalak P Noor MAF Genome-wide patterns of expression in *Drosophila *pure-species and hybrid males Mol Biol Evol 2003 20 1070 1076 10.1093/molbev/msg119 12777520

[B18] Ranz JM Castillo-Davis CI Meiklejohn CD Hartl DL Sex-dependent gene expression and evolution of the *Drosophila *transcriptome Science 2003 300 1742 1745 10.1126/science.1085881 12805547

[B19] Becher M Talke IN Krall L Krämer U Cross-species microarray transcript profiling reveals high constitutive expression of metal homeostasis genes in shoots of the zinc hyperaccumulator *Arabidopsis halleri* Plant J 2004 37 251 268 14690509 10.1046/j.1365-313x.2003.01959.x

[B20] Close TJ Wanamaker SI Caldo RA Turner SM Ashlock DA Dickerson JA Wing RA Muehlbauer GJ Kleinhofs A Wise RP A new resource for cereal genomics: 22 K barley genechip comes of age Plant Physiol 2004 134 960 968 10.1104/pp.103.034462 15020760 PMC389919

[B21] Ji W Zhou W Gregg K Yu N Davis S Davis S A method for cross-species gene expression analysis with high-density oligonucleotide arrays Nucleic Acids Res 2004 32 e93 10.1093/nar/gnh084 15247326 PMC443552

[B22] Khaitovich P Weiss G Lachmann M Hellmann I Enard W Muetzel B Wirkner U Ansorge W Pääbo S A neutral model of transcriptome evolution PLoS Biology 2004 2 682 689 10.1371/journal.pbio.0020132 PMC406393 15138501

[B23] Nuzhdin SV Wayne ML Harmon KL McIntyre LM Common pattern of evolution of gene expression level and protein sequence in *Drosophila* Mol Biol Evol 2004 21 1308 1317 10.1093/molbev/msh128 15034135

[B24] Uddin M Wildman DE Liu G Xu W Johnson RM Hof PR Sister grouping of chimpanzees and humans as revealed by genome-wide phylogenetic analysis of brain gene expression profiles Proc Natl Acad Sci USA 2004 101 2957 2962 10.1073/pnas.0308725100 14976249 PMC365727

[B25] Weber M Harada E Vess C Roepenack-Lahaye E Clemens S Comparative microarray analysis of *Arabidopsis thaliana *and *Arabidopsis halleri *roots identifies nicotianamine synthase, a ZIP transporter and other genes as potential metal hyperaccumulation factors Plant J 2004 37 269 281 10.1111/j.1365-313X.2003.02013.x 14690510

[B26] Wang Z Lewis MG Nau ME Arnold A Vahey MT Identification and utilization of inter-species conserved (ISC) probesets on Affymetrix human GeneChip^® ^platforms for the optimization of the assessment of expression patterns in non human primate (NHP) samples BMC Bioinformatics 2004 5 1 165 10.1186/1471-2105-5-165 15507140 PMC526766

[B27] Hammond JP Broadley MR Craigon DJ Higgins J Emmerson ZF Townsend HJ White PJ May ST Using genomic DNA-based probe-selection to improve the sensitivity of high-density oligonucleotide arrays when applied to heterologous species Plant Methods 2005 1 10 10.1186/1746-4811-1-10 16280083 PMC1308859

[B28] Moore S Payton P Wright M Tanksley S Giovannoni J Utilization of tomato microarrays for comparative gene expression analysis in the Solanaceae J Exp Bot 2005 56 2885 2895 10.1093/jxb/eri283 16216847

[B29] Vallée M Robert C Méthot S Palin M-F Sirard M-A Cross-species hybridizations on a multi-species cDNA microarray to identify evolutionarily conserved genes expressed in oocytes BMC Genomics 2006 7 113 10.1186/1471-2164-7-113 16686947 PMC1475851

[B30] Hammond JP Bowen HC White PJ Mills V Pyke KA Baker AJM Whiting SN May ST Broadley MR A comparison of the *Thlaspi caerulescens *and *Thlaspi arvense *shoot transcriptomes New Phytologist 2006 170 239 260 10.1111/j.1469-8137.2006.01662.x 16608451

[B31] Nieto-Díaz M Pita-Thomas W Nieto-Sampedro M Cross-species analysis of gene expression in non-model mammals:reproducibility of hybridization on high density oligonucleotide microarrays BMC Genomics 2007 8 89 10.1186/1471-2164-8-89 17407579 PMC1853087

[B32] Chain FJJ Ilieva D Evans BJ Single-species microarrays and comparative transcriptomics PLoS ONE 2008 3 9 e3279 10.1371/journal.pone.0003279 18815615 PMC2533705

[B33] Irizarry RA Bolstad BM Collin F Cope LM Hobbs B Speed TP Summaries of Affymetrix GeneChip probe level data Nucleic Acids Res 2003 31 e15 10.1093/nar/gng015 12582260 PMC150247

[B34] Borevitz JO Liang D Plouffe D Chang H-S Zhu T Weigel D Berry CC Winzeler E Chory J Large-scale identification of single-feature polymorphisms in complex genomes Genome Res 2003 13 513 523 10.1101/gr.541303 12618383 PMC430246

[B35] Walter NAR McWeeney SK Peters ST Belknap JK Hitzemann R Buck KJ SNPs matter: impact on detection of differential expression Nature Methods 2007 4 679 680 10.1038/nmeth0907-679 17762873 PMC3410665

[B36] DeCook R Lall S Nettleton D Howell SH Genetic regulation of gene expression during shoot development in Arabidopsis Genetics 2006 172 1155 1164 10.1534/genetics.105.042275 15956669 PMC1456214

[B37] Thimm O Bläsing O Gibon Y Nagel A Meyer S Krüger P Selbig J Müller LA Rhee SY Stitt M MAPMAN: a user-driven tool to display genomics data sets onto diagrams of metabolic pathways and other biological processes Plant J 2004 37 914 939 10.1111/j.1365-313X.2004.02016.x 14996223

[B38] Usadel B Nagel A Steinhauser D Gibon Y Bläsing OE Redestig H Sreenivasulu N Krall L Hannah MA Poree F Fernie AR Stitt M PageMan:An interactive ontology tool to generate, display, and annotate overview graphs for profiling experiments BMC Bioinformatics 2006 7 535 10.1186/1471-2105-7-535 17176458 PMC1766370

[B39] Demura T Fukuda H Transcriptional regulation in wood formation Trends Plant Sci 2007 12 64 70 10.1016/j.tplants.2006.12.006 17224301

[B40] Kubo M Udagawa M Nishikubo N Horiguchi G Yamaguchi M Ito J Mimura T Fukuda H Demura T Transcription switches for protoxylem and metaxylem vessel formation Genes Dev 2005 19 1855 1860 10.1101/gad.1331305 16103214 PMC1186185

[B41] Mitsuda N Seki M Shinozaki K Ohme-Takagi M The NAC transcription factors NST1 and NST2 of *Arabidopsis *regulate secondary wall thickenings and are required for anther dehiscence Plant Cell 2005 17 2993 3006 10.1105/tpc.105.036004 16214898 PMC1276025

[B42] Mitsuda N Iwase A Yamamoto H Yoshida M Seki M Shinozaki K Ohme-Takagi M NAC transcription factors, NST1 and NST3, are key regulators of the formation of secondary walls in woody tissues of *Arabidopsis* Plant Cell 2007 19 270 280 10.1105/tpc.106.047043 17237351 PMC1820955

[B43] Zhong R Demura T Ye Z-H SND1, a NAC domain transcription factor, is a key regulator of secondary wall synthesis in fibers of *Arabidopsis* Plant Cell 2006 18 3158 3170 10.1105/tpc.106.047399 17114348 PMC1693950

[B44] Zhong R Richardson EA Ye Z-H The MYB46 transcription factor is a direct target of SND1 and regulates secondary wall biosynthesis in *Arabidopsis* Plant Cell 2007 19 2776 2792 10.1105/tpc.107.053678 17890373 PMC2048704

[B45] Zhong R Lee C Zhou J McCarthy RL Ye Z-H A battery of transcription factors involved in the regulation of secondary cell wall biosynthesis in *Arabidopsis* Plant Cell 2008 20 2763 2782 10.1105/tpc.108.061325 18952777 PMC2590737

[B46] Yang C Xu Z Song J Conner K Barrena GV Wilson ZA *Arabidopsis MYB26/MALE STERILE35 *regulates secondary thickening in the endothecium and is essential for anther dehiscence Plant Cell 2007 19 534 548 10.1105/tpc.106.046391 17329564 PMC1867336

[B47] Zhou J Lee C Zhong R Ye Z-H MYB58 and MYB63 are transcriptional activators of the lignin biosynthetic pathway during secondary cell wall formation in *Arabidopsis* Plant Cell 2009 21 248 266 10.1105/tpc.108.063321 19122102 PMC2648072

[B48] Kawaoka A Kaothien P Yoshida K Endo S Yamada K Ebinuma H Functional analysis of tobacco LIM protein Ntlim1 involved in lignin biosynthesis Plant J 2000 22 289 301 10.1046/j.1365-313x.2000.00737.x 10849346

[B49] Rogers LA Campbell MM The genetic control of lignin deposition during plant growth and development New Phytol 2004 164 17 30 10.1111/j.1469-8137.2004.01143.x 33873487

[B50] Goicoechea M Lacombe E Legay S Mihaljevic S Rech P Jauneau A Lapierre C Pollet B Verhaegen D Chaubet-Gigot N Grima-Pettenati J *Eg*MYB2, a new transcriptional activator from *Eucalyptus *xylem, regulates secondary cell wall formation and lignin biosynthesis Plant J 2005 43 553 567 10.1111/j.1365-313X.2005.02480.x 16098109

[B51] Shiu S-H Bleecker AB Receptor-like kinases from *Arabidopsis *form a monophyletic gene family related to animal receptor kinases Proc Natl Acad Sci USA 2001 98 10763 10768 10.1073/pnas.181141598 11526204 PMC58549

[B52] Shiu S-H Karlowski WM Pan R Tzeng Y-H Mayer KFX Li W-H Comparative analysis of the receptor-like kinase family in Arabidopsis and rice Plant Cell 2004 16 1220 1234 10.1105/tpc.020834 15105442 PMC423211

[B53] Afzal AJ Wood AJ Lightfoot DA Plant receptor-like serine threonine kinases: Roles in signaling and plant defense Molecular Plant-Microbe Interactions 2008 21 507 517 10.1094/MPMI-21-5-0507 18393610

[B54] Hématy K Sado P-E Van Tuinen A Rochange S Desnos T Balzergue S Pelletier S Renou J-P Höfte H A receptor-like kinase mediates the response of *Arabidopsis *cells to the inhibition of cellulose synthesis Curr Biol 2007 17 922 931 10.1016/j.cub.2007.05.018 17540573

[B55] Xu S-L Rahman A Baskin TI Kieber JJ Two leucine-rich repeat receptor kinases mediate signalling, linking cell wall biosynthesis and ACC synthase in *Arabidopsis* Plant Cell 2008 20 3065 3079 10.1105/tpc.108.063354 19017745 PMC2613664

[B56] He Z-H Fujiki M Kohorn BD A cell wall-associated, receptor-like protein kinase J Biol Chem 1996 271 19789 19793 10.1074/jbc.271.33.19789 8702686

[B57] Anderson CM Wagner TA Perret M He Z-H He D Kohorn BD WAKs: cell wall-associated kinases linking the cytoplasm to the extracellular matrix Plant Mol Biol 2001 47 197 206 10.1023/A:1010691701578 11554472

[B58] Turner SR Somerville CR Collapsed xylem phenotype of *Arabidopsis *identifies mutants deficient in cellulose deposition in the secondary cell wall Plant Cell 1997 9 689 701 10.1105/tpc.9.5.689 9165747 PMC156949

[B59] Taylor NG Scheible W-R Cutler S Somerville CR Turner SR The *irregular xylem3 *locus of *Arabidopsis *encodes a cellulose synthase required for secondary cell wall synthesis Plant Cell 1999 11 769 779 10.1105/tpc.11.5.769 10330464 PMC144224

[B60] Taylor NG Laurie S Turner SR Multiple cellulose synthase catalytic subunits are required for cellulose synthesis in *Arabidopsis* Plant Cell 2000 12 2529 2539 10.1105/tpc.12.12.2529 11148295 PMC102235

[B61] Taylor NG Howells RM Huttly AK Vickers K Turner SR Interactions among three distinct CesA proteins essential for cellulose synthesis Proc Natl Acad Sci USA 2003 100 1450 1455 10.1073/pnas.0337628100 12538856 PMC298793

[B62] Liepman AH Wilkerson CG Keegstra K Expression of cellulose synthase-like (Csl) genes in insect cells reveals that CslA family members encode mannan synthases Proc Natl Acad Sci USA 2005 102 2221 2226 10.1073/pnas.0409179102 15647349 PMC548565

[B63] Jung H-G Engels FM Alfalfa stem tissues: Cell-wall deposition, composition, and degradability Crop Sci 2002 42 524 534

[B64] Brouwer DJ Osborn TC A molecular marker linkage map of tetraploid alfalfa (*Medicago sativa *L.) Theor Appl Genet 1999 99 1194 1200 10.1007/s001220051324

[B65] Choi H-K Mun J-H Kim D-J Zhu H Baek J-M Mudge J Roe B Ellis N Doyle J Kiss GB Young ND Cook DR Estimating genome conservation between crop and model legume species Proc Natl Acad Sci USA 2004 101 15289 15294 10.1073/pnas.0402251101 15489274 PMC524433

[B66] Choi H-K Kim D Uhm T Limpens E Lim H Mun J-H Kalo P Penmetsa RV Seres A Kulikova O Roe BA Bisseling T Kiss GB Cook DR A sequence-based genetic map of *Medicago truncatula *and comparison of marker colinearity with *M. sativa* Genetics 2004 166 1463 1502 10.1534/genetics.166.3.1463 15082563 PMC1470769

[B67] Grant D Cregan P Shoemaker RC Genome organization in dicots: Genome duplication in *Arabidopsis *and synteny between soybean and *Arabidopsis* Proc Natl Acad Sci 2000 97 4168 4173 10.1073/pnas.070430597 10759555 PMC18185

[B68] Zhan S Horrocks J Lukens LN Islands of co-expressed neighboring genes in *Arabidopsis thaliana *suggest higher-order chromosome domains Plant J 2006 45 347 357 10.1111/j.1365-313X.2005.02619.x 16412082

[B69] Lercher MJ Blumenthal T Hurst LD Coexpression of neighboring genes in *Caenorhabditis elegans *is mostly due to operons and duplicate genes Genome Res 2003 13 238 243 10.1101/gr.553803 12566401 PMC420373

[B70] Hurst LD Williams EJB Pál C Natural selection promotes the conservation of linkage of co-expressed genes Trends Genetics 2002 18 12 604 606 10.1016/S0168-9525(02)02813-5 12446137

[B71] Oliver B Parisi M Clark D Gene expession neighborhoods J Biol 2002 1 1 4 10.1186/1475-4924-1-4 12144705 PMC117247

[B72] Vogel JH Von Heydebreck A Purmann A Sperling S Chromosomal clustering of a human transcriptome reveals regulatory background BMC Bioinformatics 2005 6 230 10.1186/1471-2105-6-230 16171528 PMC1261156

[B73] Kosak ST Scalzo D Alworth SV Li F Palmer S Enver T Lee JSJ Groudine M Coordinate gene regulation during hematopoiesis is related to genomic organization PLoS Biology 2007 5 11 2602 2613 10.1371/journal.pbio.0050309 PMC2080650 18031200

[B74] Michalak P Coexpression, coregulation, and cofunctionality of neighboring genes in eukaryotic genomes Genomics 2008 91 3 243 248 10.1016/j.ygeno.2007.11.002 18082363

[B75] Williams EJB Bowles DJ Coexpression of neighboring genes in the genome of *Arabidopsis thaliana* Genome Res 2004 14 1060 1067 10.1101/gr.2131104 15173112 PMC419784

[B76] Ren X-Y Fiers MWEJ Stiekema WJ Nap J-P Local coexpression domains of two to four genes in the genome of Arabidopsis Plant Physiol 2005 138 923 934 10.1104/pp.104.055673 15923337 PMC1150408

[B77] Ren X-Y Stiekema WJ Nap J-P Local coexpression domains in the genome of rice show no microsynteny with Arabidopsis domains Plant Mol Biol 2007 65 205 217 10.1007/s11103-007-9209-0 17641976 PMC2039854

[B78] Altschul SF Gish W Miller W Myers EW Lipman DJ Basic local alignment search tool J Mol Biol 1990 215 403 410 2231712 10.1016/S0022-2836(05)80360-2

