# Supplementary material for: Transcript profiling of two alfalfa genotypes with contrasting cell wall composition in stems using a cross-species platform: optimizing analysis by masking biased probes
Source: BMC Genomics. 2010 May 24;11:323. doi: 10.1186/1471-2164-11-323 (PMC2893600; doi:10.1186/1471-2164-11-323)
Supplement: Additional file 4 — Clustering simulation analysis of co-regulated genes. A table showing the result of clustering simulation analysis of co-regulated genes with a 100 kb window. [file 1471-2164-11-323-S4.DOC]

**Additional data file 4. Clustering simulation analysis of co-regulated genes (100 kb window).**

| Gene | Tissue | Number of | Simulation | Simulation | Experimental | SD from |
| --- | --- | --- | --- | --- | --- | --- |
| Category | Type | Genes^†^ | Mean^‡^ | SD | Data^§^ | Simulation Mean^¶^ |
| up in 252 | ES | 1 | 181.9 | 6.4 | 148 | -5.3 |
|  |  | 2 | 12.4 | 3 | 23 | 3.5 |
|  |  | 3 | 1.4 | 0.7 | 3 | 2.4 |
| down in 252 | ES | 1 | 198.8 | 7.2 | 167 | -4.4 |
|  |  | 2 | 15 | 3.4 | 22 | 2.1 |
|  |  | 3 | 1.5 | 0.8 | 4 | 3.1 |
| up in 252 | PES | 1 | 280.4 | 10 | 247 | -3.4 |
|  |  | 2 | 31.6 | 4.7 | 37 | 1.2 |
|  |  | 3 | 3.2 | 1.6 | 3 | -0.2 |
|  |  | 4 | 1.1 | 0.4 | 6 | 13.3 |
| down in 252 | PES | 315.6 | 11.3 | 279 | 307 | 1.1 |
|  |  | 41.2 | 5.4 | 52 | 46 | 0.8 |
|  |  | 4.7 | 2.1 | 9 | 5 | 0.3 |

*Genes up- or down-regulated in genotype 252 compared to genotype 1283

^†^Number of genes identified within the 50 kb bin of the simulation.

^‡^Average number of bins with the corresponding gene numbers from replicated simulation (x2000)

^§^Number of 50 kb bins with the corresponding gene number from this study.

^¶^(Experimental Data – Simulation Mean)/(Simulation SD). Value of 2 or greater indicates statistically significant clustering of genes.
